# The Hematopoietic Bone Marrow Niche Ecosystem

**DOI:** 10.3389/fcell.2021.705410

**Published:** 2021-07-22

**Authors:** Julia Fröbel, Theresa Landspersky, Gülce Percin, Christina Schreck, Susann Rahmig, Alessandro Ori, Daniel Nowak, Marieke Essers, Claudia Waskow, Robert A. J. Oostendorp

**Affiliations:** ^1^Immunology of Aging, Leibniz Institute on Aging – Fritz Lipmann Institute, Jena, Germany; ^2^School of Medicine, Department of Internal Medicine III, Technical University of Munich, Munich, Germany; ^3^Proteomics of Aging, Leibniz Institute on Aging – Fritz Lipmann Institute, Jena, Germany; ^4^Department of Hematology and Oncology, Medical Faculty Mannheim, Heidelberg University, Mannheim, Germany; ^5^Heidelberg Institute for Stem Cell Technology and Experimental Medicine (HI-STEM gGmbH), Heidelberg, Germany; ^6^Division Inflammatory Stress in Stem Cells, German Cancer Research Center (DKFZ), Heidelberg, Germany; ^7^Institute of Biochemistry and Biophysics, Faculty of Biological Sciences, Friedrich Schiller University Jena, Jena, Germany; ^8^Department of Medicine III, Technical University Dresden, Dresden, Germany

**Keywords:** niche, microenvironment, bone marrow, hematopoiesis, leukemia, aging, transplantation, xenograft

## Abstract

The bone marrow (BM) microenvironment, also called the BM niche, is essential for the maintenance of fully functional blood cell formation (hematopoiesis) throughout life. Under physiologic conditions the niche protects hematopoietic stem cells (HSCs) from sustained or overstimulation. Acute or chronic stress deregulates hematopoiesis and some of these alterations occur indirectly via the niche. Effects on niche cells include skewing of its cellular composition, specific localization and molecular signals that differentially regulate the function of HSCs and their progeny. Importantly, while acute insults display only transient effects, repeated or chronic insults lead to sustained alterations of the niche, resulting in HSC deregulation. We here describe how changes in BM niche composition (ecosystem) and structure (remodeling) modulate activation of HSCs *in situ*. Current knowledge has revealed that upon chronic stimulation, BM remodeling is more extensive and otherwise quiescent HSCs may be lost due to diminished cellular maintenance processes, such as autophagy, ER stress response, and DNA repair. Features of aging in the BM ecology may be the consequence of intermittent stress responses, ultimately resulting in the degeneration of the supportive stem cell microenvironment. Both chronic stress and aging impair the functionality of HSCs and increase the overall susceptibility to development of diseases, including malignant transformation. To understand functional degeneration, an important prerequisite is to define distinguishing features of unperturbed niche homeostasis in different settings. A unique setting in this respect is xenotransplantation, in which human cells depend on niche factors produced by other species, some of which we will review. These insights should help to assess deviations from the steady state to actively protect and improve recovery of the niche ecosystem *in situ* to optimally sustain healthy hematopoiesis in experimental and clinical settings.

## Introduction: Scope of This Review

The bone marrow (BM) niche is an important gate-keeper in the maintenance of the blood cell system, which is unique in its circulation throughout the body. Blood cells are produced from a limited number of hematopoietic stem cells (HSCs), which reside in the bone marrow. It is here, that the BM microenvironment exerts its regulatory activity on HSCs and their progeny. The BM comprises a complex microenvironment of different cell types (hematopoietic and non-hematopoietic), together with extracellular components such as the extracellular matrix (ECM), chemical, and physical factors. The cells and factors coordinately regulate the balance between HSC quiescence and activation and the subsequent processes of cell fate determination, proliferation, self-renewal, and differentiation. Consequently, deregulation of BM niche cells is sufficient to interfere with normal stem cell behavior, which may even result in malignant transformation (Walkley et al., 2007a, b; Raaijmakers et al., 2010; Kode et al., 2014). Malignant cells further aggravate deregulation of normal hematopoiesis by creating their own environment which does not support efficient normal hematopoiesis (Colmone et al., 2008; Waclawiczek et al., 2020).

Deregulation of the bone marrow architecture occurs in both acute and chronic infections as well as aging (Mawatari et al., 1999; Chan and Duque, 2002; Polzer et al., 2010; Hirche et al., 2017). Such morphologic age-related changes are further observed in chronic malignancies, such as myelodysplastic syndrome (MDS), and myeloproliferative neoplasms (MPNs), which are consistently found together with ineffective normal hematopoiesis (Bartl et al., 1992). During ageing as well as in chronic malignancies these morphological changes sometimes co-inside with a prominent increase in an inflammatory milieu (Bartl et al., 1992; Hasselbalch, 2013), underscoring the role for inflammatory processes in the development of the changes. Still, it remains poorly understood which precise cells or cellular units within the niche are responsible for the deregulation of quiescent, healthy HSCs (Waclawiczek et al., 2020), and promote survival or accumulation of malignant clones (Lutzny et al., 2013).

Together, these studies not only highlight the complexity of the BM niche, they also provide opportunities for the BM microenvironment as a therapeutic target to interfere with microenvironmental support in pathological states, or to improve healthy hematopoiesis. Furthermore, these studies illustrate that the BM niche can only be understood through integrated approaches looking at the tissue as an adaptable ecosystem (Schmitt-Graeff et al., 2015).

Here, we will review how the BM maintains HSCs by approaching the BM, HSCs and their progeny as such an ecosystem. This means that we will not discuss the wealth of data gathered from *in vitro* cell culture studies, for which we would like to refer to a bioinformatic integration of bulk RNAseq analyses of different cell lines and sorted BM cells known to maintain HSCs in *in vitro* co-cultures (Desterke et al., 2020). Bearing in mind the wealth of data concerning *in vivo* BM niche dependence in normal, stressed, and malignant hematopoiesis, but the scarcity of precise data about the numbers and behavior of the exact cell types involved, we will review how stress stimuli or malignant disease alters niche heterogeneity in experimental systems and patients in brief summaries. We will first focus on the different cell types and then highlight some secreted factors that have been shown to be important for human hematopoiesis in experimental xenogeneic systems, highlighting how the murine BM niche supports hematopoiesis across species barriers.

## The Steady-State and Stress of the Bone Marrow Niche

In order to understand changes from the norm, it is critical that hallmarks of the homeostatic steady state of the healthy niche are defined. The hematopoietic niche consists of hematopoietic cells intertwined by different types of supportive cells of the microenvironment. In mammals, this cellular network is localized mainly in the BM. The cellular composition of the BM under steady-state as well as in different “stress states” has been assessed by bulk analyses and single cell approaches to investigate the heterogeneity in niche cells, by assessing their presence at defined anatomical sites, by identifying cell-cell interactions and communication based on ligand-receptor pairing, and through exploring interactions of niche cells with the ECM. In the future, these studies will help to identify a “bench mark” for what could be defined as the steady-state of a healthy BM niche in terms of which cells are present, in which numbers, and what is their steady-state transcriptome, proteome, or secretome.

Recently, single cell analyses have been instrumental in elucidating the composition of the niche as an ecology of different interdependent cell types. Analyses aimed at deciphering the composition of the murine BM niche in mice have identified over twenty different subpopulations of cells (Baryawno et al., 2019; Tikhonova et al., 2019; Baccin et al., 2020; Zhong et al., 2020) with distinct lineage relationships [designated populations P1 to P7 by Wolock et al. (2019)]. An integrated analysis combining all of the datasets in these studies defined fourteen meta-clusters of cell subpopulations characterized by the expression of pro-hematopoietic factors (Dolgalev and Tikhonova, 2021). These fourteen clusters comprise endothelial cells [ECs: arterial (AEC), arteriolar, and sinusoidal (SEC), 32% of all cells], mesenchymal stem and progenitor cells [adipogenic and osteogenic MSPCs, also designated as CXCL12-abundant reticular (CAR) cells, 27%], osteoblasts (mature and immature, 5%), chondrocytes (16%), fibroblasts (18%), pericytes (1%), smooth muscle cells (0.1%), and Schwann cells (0.3%)^1^ (Dolgalev and Tikhonova, 2021).

Since the earliest description of BM-derived mesenchymal stromal cells (MSCs), it has been recognized that stromal cells with the ability to form fibroblast-like colonies show differentiation into adipo-, chondro- and osteogenic lineages (Friedenstein et al., 1974; Pittenger et al., 1999; Bianco et al., 2008), indicating the close relationship between mesenchymal populations in the BM niche. The different progeny cells may, however, have different effects on HSCs, and it was shown that although adipocytes are an important source of SCF and thus, indispensable for maintaining HSCs (Zhou et al., 2017), they may be negative regulators in some skeletal tissues (Naveiras et al., 2009), because they inhibit formation of sinusoidal vasculature (Zhou et al., 2017). On the other hand, osteoblasts appear to be indispensable for HSC function (Calvi et al., 2003; Zhang et al., 2003). Both, adipo- and osteogenic lineages, are thought to arise from a common precursor, and, indeed, recent single cell studies show a consistent pattern of differentiation (Wolock et al., 2019), in which a multipotent subpopulation of osteoblastic markers corresponding to OsteoCAR cells (Dolgalev and Tikhonova, 2021), differentiates into apparently committed adipo-, osteo-, and chondrogenic precursors. Lineage fate decisions toward the osteogenic lineage may well depend on epigenetic KDM4B-dependent regulation as deletion of this methyltransferase leads to bone loss and osteoporosis (Deng et al., 2021). In addition, the osteogenic factor osteolectin (CLEC11A), is required for osteogenesis (Yue et al., 2016), and promotes skeletal maintenance through its osteoblast-specific receptor ITGA11 (Kaltz et al., 2010; Shen et al., 2019). Adipogenesis, on the other hand, is regulated by estrogens, which may lead to gender- and age-specific differences in BM homeostasis (Bragdon et al., 2015). These studies strongly suggest that a disbalance between fat and bone, not only affects skeletal tissues, but also profoundly impacts on the hematopoietic niche.

Knowledge on the cellular composition and factors released in the steady-state BM niche are helpful in a number of issues that are still being intensely debated.

First, the currently accumulating data helps in assessing and understanding the remodeling response of the niche to acute or chronic stress, such as cytotoxic treatment, irradiation, inflammatory stress, stress caused by microbial infections, as well as aging or the presence of malignant disease. Such states may induce the release of pro-inflammatory cytokines such as Interferon alpha in infections, which rapidly recruit quiescent HSCs into cell cycle (Essers et al., 2009) and induce remodeling of the vascular niche (Prendergast et al., 2017).

Second, it is still unresolved whether niche remodeling is reversible after resolving stress, or whether, similar to observations in HSCs (Flach et al., 2014; Walter et al., 2015), initial damage is not completely reversible and reduces niche health and function over time. Observations in telomere dysfunctional *Terc*^–/–^ mice strongly suggest that niche function declines over time (Ju et al., 2007). In addition, in aging and leukemia, the relative contribution of the *Lepr*^+^ *Adipoq*^+^ *Kitl*^+^ AdipoCAR population changes [down in leukemia (Baryawno et al., 2019), up in aging (Zhong et al., 2020)], thereby changing the factors to which HSCs are exposed, such as varying concentrations of ANG-1/2, CXCL12 (Stromal-Derived Factor 1: SDF1), IL7, KITL (Stem Cell Factor: SCF), and SFRP1. Observations that deletion of the *Angpt1*, *Kitl*, or *Sfrp1* genes have a strong negative impact on HSC maintenance (Renström et al., 2009; Ding et al., 2012; Zhou et al., 2015) underline the importance of AdipoCAR cells and their secreted factors in maintaining HSCs. Thus, a better understanding of the steady state niche may help to define targets useful for restoring the compromised niche in leukemia back to steady state.

Third, even under steady-state conditions, levels of HSC and the release of blood cells from the marrow shows circadian day-night oscillations, which at least in part depends on oscillating production of CXCL12 by the niche (Méndez-Ferrer et al., 2008). Interestingly, additional circadian oscillations were noted in norepinephrine and TNF levels, which appear to drive temporary increases in melatonin and ROS levels in the hematopoietic compartment (Golan et al., 2018). The melatonin not only promotes HSC self-renewal (Golan et al., 2018), but also protects stromal niche cells against toxic side-effects of ROS (Mehrzadi et al., 2017). These observations show that hematopoiesis and the niche reversibly oscillate between stressed (increases in TNF, ROS) and non-stressed states on a daily basis and suggest an important role for melatonin in protecting the niche from ROS-dependent stress.

Fourth, the above data may help to resolve the puzzling observation that induced niche remodeling in which specific niche cell populations are deleted by using iDTR/diphtheria toxin targeting, such as mesenchymal *Nes*^+^, *Lepr*^+^ or *Cspg4*^+^ (NG2^+^) cells (Méndez-Ferrer et al., 2008; Kunisaki et al., 2013; Acar et al., 2015), *Cxcl4*^+^ megakaryocytes (Bruns et al., 2014; Zhao et al., 2014), or *Gfap*^+^ Schwann cells (Yamazaki et al., 2011), all lead to a declining support of hematopoiesis and, over time, to HSC attrition and BM failure. These observations indicate that different populations within the niche are collectively responsible for maintaining HSC function over time.

Finally, although the precise architecture of the niche ecosystem is still a matter of debate, it is clear that the bone marrow architecture is essential for the integrity of HSC behavior. Indeed, precise localization within the marrow with respect to different niche cell types has a strong impact on HSC behavior, particularly on HSC quiescence (Lo Celso et al., 2009; Fujisaki et al., 2011; Haltalli et al., 2020). Importantly, recent evidence shows that HSCs localizing to perisinusoidal niches, which include megakaryocytes (Bruns et al., 2014; Zhao et al., 2014) and AdipoCAR cells (Baccin et al., 2020), are protected from aging-associated attrition (Saçma et al., 2019). This suggests that even under adverse conditions, there are anatomical regions, such as the perisinuses, which preserve HSCs and their function.

The above studies include markers that can be used to monitor and predict the extent of adverse changes from the steady-state norm and may document shifts in the pools of BM niche subpopulations and their transcriptomes/secretomes. These will be key tools to diagnose changes in niche fidelity and follow therapeutic success and prognosis upon targeting the niche.

## Long-Term HSC Maintenance by Balancing Niche Fitness and Cellular Collaboration

The mechanisms governing stem cell activation and return to quiescence still need to be resolved in detail. For instance, it is not understood why depletion of different niche cell populations all lead to the same outcome: a loss of quiescent HSCs (Méndez-Ferrer et al., 2008; Yamazaki et al., 2011; Kunisaki et al., 2013; Bruns et al., 2014; Zhao et al., 2014; Acar et al., 2015). A recently proposed competition model of a niche ecology may offer new approaches to this problem. The mathematical model predicts that changes in the composition of niche cells by default also change the degree of cooperation between those cells. As a result, the fitness of the ecosystem as a whole decreases and aging-like degeneration of the system is inevitable (Nelson and Masel, 2017). This fitness/cooperation model also provides an explanation of why there appears to be no redundancy between different BM niche subpopulations.

What hampers the study of fitness and cooperation in the BM niche is that current knowledge about non-hematopoietic populations of the hematopoietic niche is insufficient to determine how stress responses affect the fitness of these subpopulations or their ability to cooperate with other cells. Indeed, in most hematopoietic conditions, bulk and single cell analyses using different “omics” (transcript-, prote-, methyl-, or metabolomics, etc.), focus on the study of the hematopoietic cells, not on the niche cells. Thus, in order to attempt restoration of the steady state situation within the BM niche, several questions need to be answered. First, what is the optimal steady state configuration of the BM microenvironment favoring maintenance of healthy hematopoiesis. Second, niche-intrinsic factors determining fitness of different niche cell subpopulations need to be defined. Third, cellular communication between niche subpopulations need to be assessed, and finally, the degree of deviation from the steady state condition in situations of chronic stress, aging or in the presence of age-related chronic malignancies should be addressed in future studies.

## The Response of the BM Niche to Acute Stress

It is becoming clear that different stressful challenges such as LPS or polyInosine- polyCytosine (pIpC), which simulate bacterial or viral infections, respectively, lead to different outcomes. Both treatments are known to rapidly recruit quiescent HSCs into cell cycle (Walter et al., 2015; Takizawa et al., 2017), but their effect on different niche cell populations was unclear. Upon LPS treatment, HSCs start proliferation and neutrophils depart the BM, which is accompanied by remodeling of the sinusoidal vasculature (Vandoorne et al., 2018). Stromal cells are instrumental in the activation of HSCs, for example via transfer of mitochondria from stromal cells (Mistry et al., 2019). RNAseq analyses of Sca1^–^ CAR cells, PDGFRa^+^Sca1^+^ PaS cells, and either CD31^+^CD105^*hi*^ sinusoidal or CD31^*hi*^CD105^+^ arterial endothelial cells (SECs and AECs) from LPS and pIpC challenged mice, demonstrate a shift of the transcriptome of different cell types toward a proinflammatory signature, characterized by upregulation of cytokines and chemokines. Thus, IL6, CCL5, and CXCL10 are upregulated in CAR cells and CCN1 and −2, CCL2 and −7 as well as IL15 are expressed at a higher level in SECs (Helbling et al., 2019).

The question of stress-induced changes in the niche can be answered in single cell analyses, which not only show transcriptional changes, but also whether remodeling of the abundance in different niche cell populations occurs. For instance, treatment with 5-fluorouracil (5FU), which targets cells in S-phase and is frequently used to activate recruitment of quiescent HSCs into cell cycle, has long since been known to increase the frequency of niche cells with bone nodule-forming potential (Falla et al., 1993). Tikhonova and coworkers showed similar increases in AdipoCAR cells, which may perhaps be induced to differentiate along the osteogenic lineage (Wolock et al., 2019), and mature *Col16a1*^+^*Tnn*^+^ osteoblasts, while immature *Bglap*^+^*Car3*^+^ osteoblasts were decreased (Tikhonova et al., 2019). With ongoing or chronic stress, such as in mice with leukemia, most stromal cell populations are diminished, with loss of expression of quiescence-promoting genes (*Cxcl12*, *Angpt1*, *Vcam1*) in *Lepr*^+^ AdipoCAR cells (Baryawno et al., 2019). At the same time, sinusoid vessels show significantly increased diameter with reduced EC cellularity (Saçma et al., 2019), thus altering how they affect HSCs through their production of KITL [SCF (Waskow et al., 2009)], DLL1/4 or JAG2 (Saçma et al., 2019; Tikhonova et al., 2019).

Stromal cells of the BM niche are very sensitive to total body irradiation (Chamberlin et al., 1974; Abbuehl et al., 2017), which was recently supported by single cell studies (Severe et al., 2019). Intriguingly, the stromal compartment also shows a remarkably rapid recovery with an increase in osteoblast-lineage cells driven by the replacement of surviving megakaryocytes to the endosteal surface (Dominici et al., 2009; Olson et al., 2013). The expanding osteoblastic cells then promote engraftment of subsequently transplanted HSCs (Marino et al., 2013). However, the early recovery of osteoblastic cells comes at the expense of “sinusoid dilatation and congestion,” followed by a progressive decrease in cellularity and later fat degeneration (Huang et al., 2009). Although irradiation targets S-phase cells like 5FU treatment, irradiation appears to target different niche cell populations, as most niche subpopulations show severe reductions in numbers, including AdipoCAR cells and mature osteoblasts. Interestingly, however, a small CD73^+^NGFR^+^ chondrocytic population is preserved (Severe et al., 2019). Subsequent experiments with *Cd73*^–/–^ mice strongly suggest that these stress-primed cells are critical in recovering from stress responses through high expression of several pro-hematopoietic cytokines (CXCL12, IL7, KITL, SPP1, and TGFb), suggesting that monitoring CD73^+^NGFR^+^ cells may be useful in predicting recovery after irradiation.

Viral infections and pIpC both rapidly and strongly upregulate production of interferon alpha (IFNa), which, in *in vivo* stimulation studies recruit HSCs into cell cycle in both autonomous and non-autonomous mechanisms (Essers et al., 2009; Uckelmann et al., 2016; Hirche et al., 2017; Prendergast et al., 2017). Whether the non-autonomous effects involve non-hematopoietic cells remains to be established. It is clear, however, that IFNa and plpC treatments increase Sca1-dependent signaling in HSCs and the relative numbers of ECs in the BM, again through both direct and indirect mechanisms (Prendergast et al., 2017). Gene expression shows that IFNa also increases VEGF expression, suggesting the EC proliferation noted may be stimulated through this growth factor. RNAseq of SECs after pIpC treatment show a particular upregulation of *Ifnar2* and *Ifngr1*, suggesting heightened sensitivity toward interferons (Abbuehl et al., 2017). In infections with either vesicular stomatitis virus or murine cytomegalovirus HSC activation occurs, but involvement of BM niche cells remains to be elucidated (Hirche et al., 2017).

**FIGURE 1:**
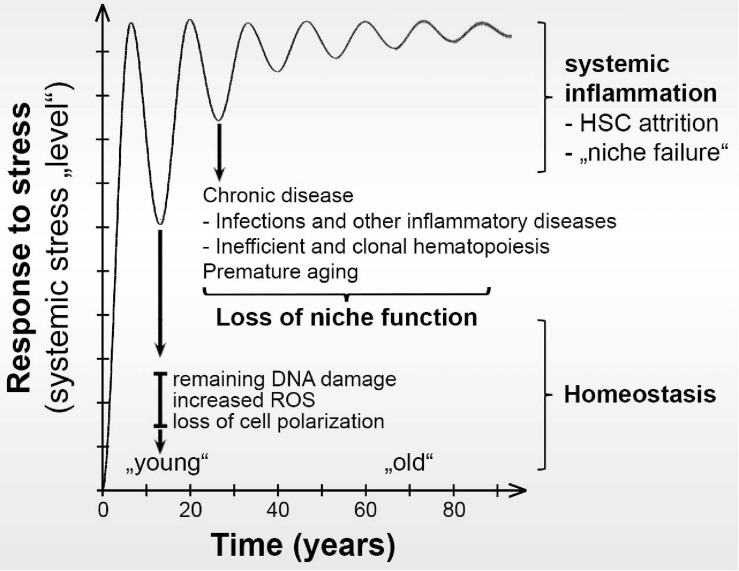
Hypothesis of how repeated stress may progressively change the fitness of BM niche cells. After each cycle of stress, some damage remains. This low frequency changes the transcriptional, proteomic, and ecological BM niche cellular composition landscapes over time. Eventually, aging-like changes in the BM niche result in loss of functional HSCs, systemic inflammation, and development of chronic alterations with loss of niche support for healthy hematopoiesis in the BM. Instead, the altered niche supports the development of chronic malignancies.

Together, these studies show that different stressors induce HSC activation and emergency hematopoiesis, which appears to depend on both changes in numbers of different niche cell subpopulations (remodeling) as well as alterations in their transcriptomes in the BM niche ecosystem (Figure 1). Most studies also show that upon a single challenge with a stressor such as LPS, pIpC, 5FU or irradiation both remodeling and transcriptome changes seem to be reversible. However, the impact of chronic inflammatory stress on niche remodeling as well as reversibility of these changes needs to be further investigated.

## Age-Related Changes in the BM Niche Ecosystem

Aging is a natural process of slow deterioration of cells and tissues over time. Aging is the main risk factor for functional decline of HSCs, defects in immunity, and increased occurrence of hematological abnormalities. Two striking features of hematopoiesis in aging humans are a relative increase in myeloid cells, and the occurrence of clonal hematopoiesis in 10–20% of healthy individuals aged 70 years or more. Clonal hematopoiesis is significantly associated with an increased risk of overall mortality, the development of cardiovascular diseases and tumors of the blood (Genovese et al., 2014; Jaiswal et al., 2014; Xie et al., 2014).

Recent studies have further shown that aging is also associated with chronic remodeling of the niche, which involves an increase in adipogenic cells (Sanchez-Aguilera et al., 2014). This fatty degeneration has been attributed to a decline in osteogenic cell fate determination (Nishikawa et al., 2010; Yu et al., 2018; Kato et al., 2019; Lacava et al., 2019) which switches mesenchymal precursors to an adipogenic state, further associated with an increase in stromal senescence (Gnani et al., 2019), loss of niche-forming endomucin-positive (EMCN^+^) CD31^*hi*^ “Type H” vessels (Kusumbe et al., 2014), and b3-adrenergic innervation (Maryanovich et al., 2018; Ho et al., 2019). Added complexity of this system is that niche remodeling is associated with an altered lineage output, including increased myelopoiesis, thrombopoiesis, and clonal hematopoiesis. These changes further result in reduction of myelopoiesis-suppressing osteocytes (Fulzele et al., 2013) and influx of myelopoiesis-promoting plasma cells (Pioli et al., 2019) as well as b2-adrenergic stimulated megakaryocytes (Ho et al., 2019) and increased platelet production (Davizon-Castillo et al., 2019; Frisch et al., 2019). In addition, telomere shortening in stromal cells is associated with reduced B-lymphopoiesis and reduced HSC function (Ju et al., 2007).

Thus, age-related processes support remodeling of the niche, which is associated with chronic malignancies (Figure 1), that often transform into aggressive myeloid leukemias for which there is currently no reliable cure. However, the link between niche remodeling, myeloid expansion, clonal hematopoiesis and increased susceptibility to chronic hematologic malignancies remains underexplored.

In single cell studies, in line with the observed increased adipogenesis, AdipoCAR numbers increase tremendously in 16-month-old mice and subdivide into three distinguishable Adipoq^+^Cxcl12^+^ populations. Furthermore, and osteogenic fate degeneration is accompanied by an almost complete loss of mature *Tnn*^+^ *Postn*^+^ osteoblasts (Zhong et al., 2020). Imaging of BM in old mice show remodeling of SECs and sinusoidal vessels with increased vessel diameter, while at the same time the total number of SECs reduces (Saçma et al., 2019). A surprising finding was that the vascular remodeling of the sinuses housed the most potent HSCs, showing that sinusoidal remodeling produces HSC-protective areas in the aging BM niche.

## Changes in the BM Niche and the Development of Chronic Malignancies

Chronic hematologic malignancies evolve over a period of several years showing clonal (pre-) oncogenic mutations. These mutations improve fitness through proliferative and/or anti-apoptotic advantages or through improving homing to the BM niche (Godavarthy et al., 2020). But, during the chronic stages, the mutations are not sufficient to allow cell-autonomous growth as pre-malignant cells depend on the niche for their survival. Over time, the microarchitecture of the BM niche distorts with associated osteopenic changes (Mawatari et al., 1999; Chan and Duque, 2002; Polzer et al., 2010) as shown in histological studies of MDS (Bartl et al., 1992), and MPN (Chan and Duque, 2002). The underlying mechanisms of microarchitectural distortion of the BM are not entirely clear, however, the niche has been shown to support malignant cells through inflammatory NFkB-driven stromal activation and the expression of proinflammatory factors, such as IL6 and TGFb1 (Krause et al., 2013). Indeed, cultured MSCs from MDS patients show inflammatory changes compared to healthy MSCs (Medyouf et al., 2014). Prospectively isolated non-cultured CD271^+^CD105^+^ MDS-MSCs also show a significant inflammatory signature (Chen et al., 2016) with increased adipogenic potential (Weickert et al., 2021), showing that stromal inflammation does not depend on cell culture and may be the result of BM niche remodeling.

MPN-MSCs differ from MDS-MSCs and show less senescence, increased cell cycling and also increased cell death with reduced differentiation into both adipogenic and osteogenic lineages (Sun et al., 2020). Although both observations point toward niche remodeling with proinflammatory features in MDS and MPN, the outcome of the remodeling is not easily predictable. Similar proinflammatory changes in other niche cells have not been studied in detail. But, ECs, for example, localize close to the endosteum and were shown to reduce proinflammatory transformation of stromal cells thereby improving maintenance of healthy hematopoiesis (Duarte et al., 2018). In addition, vascular permeability is strongly increased in AML, and targeting their NO/ROS production improves chemotherapeutic responses (Passaro et al., 2017). These studies support the view that understanding deregulation of specific subpopulations of the BM niche facilitates the development of relevant strategies to restore healthy hematopoiesis in chronic malignancies.

Age-related systemic inflammation through increased expression of IL1b and TNF (Davizon-Castillo et al., 2019) is a driving force in MDS (Chen et al., 2016), and MPN (Hasselbalch, 2013). For instance, in MDS, increased inflammation-associated and S100A8/A9-mediated genotoxic stress (Zambetti et al., 2016), ineffective hematopoiesis (Cheng et al., 2019), fatty degeneration with a decline of osteoblastic cells (Krevvata et al., 2014; Wenk et al., 2018), myelofibrosis (Leimkühler et al., 2021), and loss of niche-forming EMCN^*hi*^ vessels all potentially contribute to the disease. Systemic inflammation also drives stromal remodeling in MPN with downregulation of collagen genes and *Sparc* (Tripodo et al., 2017), neuroglial damage, and a decline in *Nes*^+^ MSCs in myelofibrotic MPN (Schmitt-Graeff et al., 2015). The extent of functional and numerical BM microenvironment remodeling *in vivo* is still to be elucidated. Age-related changes in Sp7^+^ (osterix) osteoprogenitors have been attributed to accumulation of DNA damage, pro-inflammatory changes, and senescent cells. These changes then lead to functional deterioration and depletion of these progenitors from the BM niche over time (Kim et al., 2017).

In MPN, constitutive activation of the NFkB pathway in niche cells promotes survival of oncogenic cells (Kleppe et al., 2018). Direct targeting of NFkB or its deregulated targets, such as PKC may thus induce cell death of malignant cells. Similarly, age-related denervation promotes development of AML and three sympathicomimetics improve both niche function and block progression of JAK2(V617F) MPN (Arranz et al., 2014). Interestingly, upregulation of S100a8 and a9 appears to be a general feature of proinflammatory changes in (pre-)malignant disease, and in a murine JAK2V16F model of myelofibrosis, the S100A8/9 inhibitor tasquinimod, ameliorates development of malignancies, suggesting that targeting this arm of proinflammatory molecules are tangible targets in malignancies such as MPN (Leimkühler et al., 2021).

Other age-related changes in the BM microenvironment are fatty degeneration, and the loss of niche-forming EMCN^*hi*^ vessels. Both characteristics are also found in MDS (Wenk et al., 2018) and AML (Duarte et al., 2018), respectively. These observations suggest that anti-adipogenic therapy, such as through inhibition of PPAR or PIM kinases may be of benefit for MDS and other chronic malignancies. The observed vessel remodeling in AML can be rescued using deferoxamine, which besides targeting iron overload also inhibits the collagen biosynthesis modulator prolyl-4-hydroxylase (P4H or PHD) (Duarte et al., 2018). Interestingly, deferoxamine is already used to treat patients with iron overload in MDS. Thus, deferoxamine may have a dual effect through normalizing effects on iron overload as well as restoring remodeling of niche-forming vessels.

## The BM Niche in Hematopoietic Transplantation and Graft-Versus-Host Disease

In transplantation protocols, the host is not only conditioned through irradiation or cytotoxic drug treatment, but also infused with donor hematopoietic cells. Conditioning reduces the residual cancer load, dampens the immune response to the graft, and is thought to “create space” for the incoming graft cells to grow in. Since both irradiation and cytotoxic treatments damage the BM niche, it remains poorly understood through which mechanisms the microenvironment affects the long-term success-rate in hematopoietic transplantation. It is clear, however, that several niche factors which do not seem to play a major role in steady-state hematopoiesis, such as, *Ptn*, *Sfrp1/2*, and *Wnt5a*, are required for successful regeneration of quiescent HSCs (Renström et al., 2009; Istvanffy et al., 2011; Ruf et al., 2016; Schreck et al., 2017). This observation indicates that the process of regeneration requires a different set of factors than maintaining HSCs during steady-state conditions. As mentioned above, it has long been thought that irradiation or cytotoxic conditioning depletes existing niches, which can then be occupied by incoming donor cells. However, evidence is accumulating that conditioning also stimulates the expression of engraftment-promoting factors. An example of this is the secretion of PTN, which, under steady-state conditions is mostly expressed by mature osteoblasts and *Lepr*^+^ cells, but after irradiation its expression also increases in expanding *Cdh5*^+^ ECs (Himburg et al., 2012, 2018). It was already established that engraftment was poorer in *Ptn*^–/–^ mice (Himburg et al., 2010; Istvanffy et al., 2011). Later studies showed that although HSC maintenance mainly depends on PTN from *Lepr*^+^ cells under steady-state conditions, expression from both cellular sources is required for successful engraftment (Himburg et al., 2018).

Another issue important for successful engraftment of HSCs is the presence of the donor cells, whose function depends on the cooperation of the graft with the BM niche. Indeed, recipient-dependent parameters, such as gender (Notta et al., 2010) and aging (McCurdy et al., 2018) have been shown to have a critical impact on engraftment. Gender-specific steroid-signaling through the estrogen receptors promotes engraftment of human HSC in immunodeficient female mice (Notta et al., 2010), possibly by promoting HSC self-renewal in female mice (Nakada et al., 2014). Estrogen signals differentially affect HSCs and progenitor cells, as it was shown that the estrogen modulator Tamoxifen induces apoptosis in more mature HSPCs (Sanchez-Aguilera et al., 2014). The effects of estrogens on the niche have not been described in detail. It is clear, however, that the estrogen receptor *Esr1* is expressed by both Adipo- and OsteoCAR cells, as well as fibroblasts (Dolgalev and Tikhonova, 2021). Thus, estrogen-directed signals affecting these cells of the niche may be expected in steady-state hematopoiesis and in transplantations.

Moreover, serious transplant-related complications, such as capillary leak syndrome or thrombotic angiopathy, affect the microenvironment through endothelial dysfunction (Pagliuca et al., 2019).

It remains to be studied to what extent donor hematopoietic cells help in restoring the niche function in transplantation. In *in vitro* co-culture experiments of HSPCs with stromal cells, it was shown that upon contact of the hematopoietic cells, the stromal cells produce Ctgf (Ccn2) which promotes HSC self-renewal (Istvanffy et al., 2015). It is unclear whether donor cells similarly alter gene expression of BM niche cells upon transplantation. Other studies have demonstrated that co-transplantation of recipient mice with either MSCs or ECs in murine transplantation models limit niche damage and prevent premature exhaustion of normal HSCs (Abbuehl et al., 2017; Poulos et al., 2017) or facilitate engraftment of HSCs from MDS patients (Medyouf et al., 2014).

One way of improving clinical outcomes is physical exercise during the revalidation phase after hematopoietic transplantation (Prins et al., 2021). Recent studies strongly suggest that the benefit of exercise is at least partly due to effects on the niche, since exercise increases the levels of CXCL12 and KITL (SCF) while lowering Leptin (Frodermann et al., 2019), an effect that is also observed in exercising middle-aged or obese patients (Rostás et al., 2017). In addition, the lowering of leptin levels reduces body fat, exercise increases a population of osteogenesis-committed *Clec11a*^+^*Lepr*^+^ cells which promote bone formation, most probably by stimulating signaling through the PIEZO1 cation channel in these cells (Shen et al., 2021).

A major complication in transplant procedures is the development of graft-versus-host disease (GvHD), which accounts for 10–20% mortality amongst transplant recipients. Acute GvHD is initiated by systemic inflammation associated with IL-1, IFN, and TNF signaling. These early events are preceded by angiogenesis with metabolic activation of ECs (Riesner et al., 2017; Furukawa et al., 2019), which could lead to EC activation syndromes (Pagliuca et al., 2019). This phase is followed by development of recipient-reactivity of donor T cells. Prolonged IFN and TNF signaling is further known to promote apoptosis of ECs. In addition, the inflammatory cytokines upregulate MHC class II, thus rendering different cell types targets for recipient-reactive donor T cells. This may have a severe effect on the composition of the BM niche in GvHD, as is clear by the finding that the number of CFU-F-forming MSCs reduces with the grade of GvHD (Okamoto et al., 1991). Such a damaged environment is not conducive to either maintaining host HSCs or engraftment by donor HSCs, which results in poor graft function, or graft failure (Masouridi-Levrat et al., 2016).

## Xenograft Models: Human HSC Engraftment in the Murine BM Niche

To link cell biological modifications to functional alterations in human HSCs (huHSCs) or to determine the contribution of niche cells to normal and malignant human hematopoiesis *in vivo*, a surrogate microenvironment is required. Thus, an interesting approach is the study of the BM niche in xenograft models. In such models, engraftment of human cells depends on the murine niche. But, not all factors expressed and produced by the host (murine) environment (cytokines, adhesion molecules, extracellular matrix) act on the (human) donor cells. Thus, xenograft models enable the definition of species-dependent and -independent cellular and molecular pathways as targets to improve niche formation/restoration as well as transplantation protocols. To prevent xenogenic donor-cell rejection and to allow for their settlement in the bone marrow niche, immune deficient animals are used to prevent graft rejection, and recipient mice need to be conditioned using irradiation or chemical treatment before transplantation similar to syn- and congenic transplantation models. However, as described above, such conditioning is toxic for sinusoidal blood vessels and MSCs in the murine stem cell niche (Yin et al., 2020) and, importantly, it impairs the function of xenogenic donor HSCs promoting their senescence and apoptosis by increasing ROS levels and mitochondrial damage (Hu et al., 2021).

To overcome this problem, we have established recipient mouse models that allow for transplantation of huHSCs in the absence of a toxic conditioning regimen. In such models, endogenous murine HSCs have a competitive disadvantage compared to donor huHSCs, and as a consequence, donor stem cells stably engraft in the murine stem cell niche (Cosgun et al., 2014; Rahmig et al., 2016). Furthermore, reduced competitiveness of endogenous murine HSCs is conferred by loss of function mutations in the receptor tyrosine kinase Kit. We have shown before that mutant *Kit* combined with immunodeficiency mediated by null mutations in the recombination activating gene 2 (*Rag2*) and interleukin 2 receptor gamma chain gene (*Il2rgc*) facilitates near-complete engraftment of murine syngeneic but also histoincompatible allogeneic donor mouse HSCs in the absence of any further conditioning therapy (Waskow et al., 2009). The C57BL/6 genetic background is not suitable for the transplantation of huHSCs because donor cells are subject to clearance by phagocytes (Takenaka et al., 2007). The introduction of mutant *Kit* onto immune deficient mice of different genetic backgrounds including BALB/c and NOD allows for efficient engraftment of xenogenic human donor HSCs and multilineage reconstitution without any need of physical conditioning therapy (Cosgun et al., 2014; Rahmig et al., 2016). The mouse lines generated are BRgWv [Balb/c.*Rag2^–/–^Il2rgc^–/–^Kit^*Wv/Wv*^;* (Cosgun et al., 2014)], BRgSK [C57BL/6.*Rag2^–/–^Il2rgc^–/–^*NOD-*Sirpa Kit*^*Wv/Wv*^ (Yurino et al., 2016)], and NSGW41 [NOD. *Prkdc^*Scid*^Il2rg^–/–^Kit^*W*41/W41^* (Cosgun et al., 2014; Rahmig et al., 2016)], and NBSGW [NOD/B6.*Prkdc^*Scid*^Il2rg^–/–^Kit^*W*41/W41^* (McIntosh et al., 2015)].

## The Factors Supporting Human HSCs in the Murine BM Niche

**FIGURE 2:**
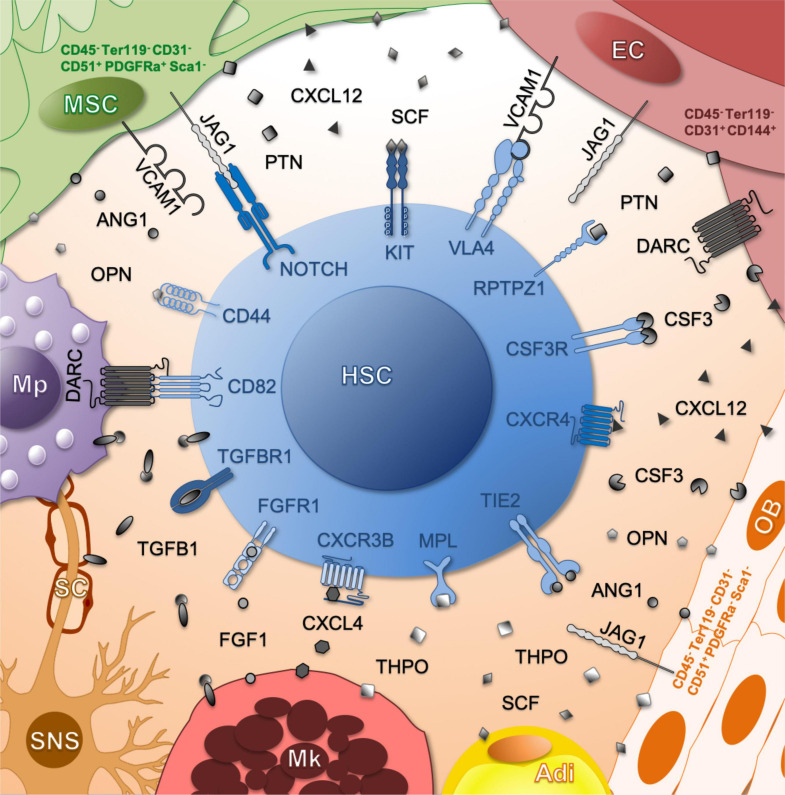
Schematic representation of molecular communication partners between HSCs and their niche. The ligands produced by each niche cell are indicated. Color of receptors correlates to the number of ligand-secreting cell types (dark – many producers, light – few producers). Adi, adipocyte; EC, endothelial cell; Mk, megakaryocyte; Mp, macrophage; MSC, mesenchymal stromal cell; Ob, osteoblast; SC, Schwann cell; SNS, sympathetic nervous system.

It remains unclear which murine niche cell types and molecular interactions promote huHSC engraftment and continued human hematopoiesis in the murine microenvironment. This is further complicated by the fact that many factors are produced by a set of distinct niche cell types and that many defined niche cells provide more than one important factor (Figure 2). We focus on factors produced by murine non-hematopoietic niche cells that have been investigated also in the context of huHSC engraftment in mice.

One of the most important and earliest identified receptor/ligand interactions crucial for hematopoietic stem and progenitor cell function is the KITL (SCF) - KIT signaling axis (McCarthy et al., 1977; Broxmeyer et al., 1991; Ikuta et al., 1991; Waskow et al., 2002). In single cell studies from steady-state BM cells, expression of *Kitl* is mostly found in *Lepr*^+^ AdipoCAR cells, AECs, some pericytes and OsteoCAR cells, whereas *Kit* is expressed in scattered AdipoCAR cells and SECs (Dolgalev and Tikhonova, 2021). Elegant, cell-type-specific depletion experiments have shown that mSCF provided by distinct niche cells can affect the function of different HSC and progenitor populations. *Kitl*-encoding transcripts are primarily detected in MSCs (Table 1) and perivascular cells are consistently labeled in *Scf-gfp* mice (Ding et al., 2012). Depletion of *Scf* in *Lepr*^+^ cells, a population that is included in CD51^+^PDGFRa^+^Sca1^–^ MSCs (Mende et al., 2019), and CD105^+^Tie2^+^ ECs results in the loss of murine HSCs *in situ* (Ding et al., 2012). Further, deletion of *Kitl* from *Lepr*^+^ cells also depletes myelo-erythroid but no other hematopoietic progenitor cells, suggesting specialization of vascular niches in supporting HSC and myeloid progenitors harboring defined differentiation propensity, particularly toward the erythroid lineage (Comazzetto et al., 2019; Zhang et al., 2021). In line with this idea, lack of mSCF produced by ECs results in the depletion of stem but not progenitor cells, further indicating spatial separation of functional niches (Comazzetto et al., 2019; Zhang et al., 2021). Consistent with a role for mSCF derived from *Lepr*^+^ cells in erythroid differentiation, the specific support of erythroid differentiation mediates survival of *Kit*-null mice where hematopoietic progenitor cells cannot respond to mSCF signals (Waskow et al., 2004), further pointing at a crucial role of mSCF-mediated signals in supporting erythroid progenitor function. Along this line, it was shown that soluble mSCF plays a significant role in myeloid progenitor production whereas its membrane-bound isoform has more importance for the erythroid lineage (Kapur et al., 1998). Further, mSCF-mediated signals have distinct effects on maintenance and proliferation of HSCs that express different levels of mKIT (Grinenko et al., 2014; Shin et al., 2014), suggesting that not only the cellular source and isoform, but also receptor density on the target cell population shapes the mutual niche/HSC communication.

**TABLE 1:** Overview of molecular interaction partners between human/mouse HSCs and mouse bone marrow niche cells.

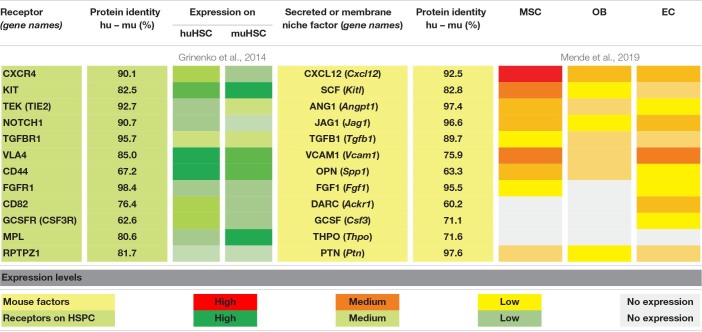

*Color intensity contains information on the amount of transcripts encoding for either receptors expressed by human/mouse HSCs (green) or factors expressed by indicated niche cells (red/yellow). Color intensity was introduced based on read counts from bulk sequencing approaches using prospectively isolated mouse (CD45/Ter119/Lin)^–^ CD31^–^ CD144^–^ CD51^+^ SCA1^–^ PDGFRa^+^ MSC or PDGFRa^–^ OB, and (CD45/Ter119/Lin)^–^ CD31^+^CD144^+^ EC (Mende et al., 2019)and human and mouse HSCs (Grinenko et al., 2014). Protein sequence identity between human and mouse receptors and ligands were calculated using BLAST implemented in UniProt (UniProt Consortium, 2021).*

In xenotransplantation experiments, we have shown that hKIT-proficient human donor HSCs engraft best into mKIT-mutant mouse recipients in the steady state (Cosgun et al., 2014). This result suggests that functional signaling through the KIT receptor provides a competitive advantage to human HSCs in the murine BM niche. Furthermore, engraftment of human erythroid and megakaryocyte progenitor populations in mKIT-mutant recipients is significantly increased compared to conventional, mKIT-proficient recipient mice (Rahmig et al., 2016). Considering the existence of a vascular *Lepr*^+^ erythropoiesis-promoting niche (Comazzetto et al., 2019), this either suggests improved central engraftment of huHSCs in the stem cell niche, or an advantage of KIT-proficient human donor progenitor cells over endogenous mKIT-mutant precursors, or a combination of both. Taken together, the data suggests that human HSCs and HPCs show better engraftment when the endogenous counterparts have diminished mKIT activity. Together with the high protein identity between human and murine KIT and SCF (Table 1), we predict that mSCF produced by murine *Lepr*^+^ MSCs is crucial for the maintenance of human hematopoietic stem and defined progenitor cells in mice.

The chemokine CXCL12 (SDF1) is the ligand of CXCR4 and the main chemoattractant for murine HSC homing and maintenance (Sugiyama et al., 2006). *Cxcl12* is expressed by various non-hematopoietic niche cells such as AECs (Ding and Morrison, 2013), osteoblasts (Ding and Morrison, 2013), and MSCs (Greenbaum et al., 2013; Asada et al., 2017; Table 1). *Cxcl12* shows the highest transcript abundance of niche factors discussed (Table 1), especially in the subset of *Lepr^+^Adipoq^+^* AdipoCAR cells (Dolgalev and Tikhonova, 2021). More than 90% of murine HSCs in the BM are in direct contact to CAR-MSCs that surround the sinusoidal endothelium (Sugiyama et al., 2006). Constitutive deletion of *Cxcr4* (Sugiyama et al., 2006) or *Cxcl12* (Tzeng et al., 2011) results in the depletion of HSCs from the BM. Conditional depletion mutants of *Cxcl12* from distinct niche cells in mice revealed that mCXCL12 expressed by *Tie2*^+^ endothelial cells and *Lepr*^+^ MSCs is required for both HSC retention and maintenance (Ding and Morrison, 2013; Greenbaum et al., 2013; Asada et al., 2017), whereas *Col2.3^+^* osteoblast-derived mCXCL12 supports the function of early lymphoid progenitors (Ding and Morrison, 2013). Interestingly, sympathetic nerves regulating circadian oscillations mediate daily fluctuations of HSC numbers mobilized from the bone marrow into the circulation in a *Cxcl12*-dependent manner (Katayama et al., 2006; Méndez-Ferrer et al., 2008; Golan et al., 2018).

CXCL12 is highly conserved between species, and human and mouse CXCL12 and its receptor CXCR4 both show a protein identity of 92.5 and 90.1% (Table 1), respectively. The proteins from both species differ in only one amino acid, suggesting cross-species compatibility. The administration of the CXCL12 antagonist AMD3100 (plerixafor), efficiently mobilizes murine and human HSPCs (Broxmeyer et al., 2005), as well as human HSCs in humanized mice (Samuelson et al., 2021). A role for the mCXCL12 - hCXCR4 signaling axis in homing of human HSCs in mice was further shown by reduced engraftment of human HSCs in NOD/SCID mice after pretreatment of donor cells with anti-CXCR4 antibodies (Peled et al., 1999). Consistently, overexpression of human CXCR4 on huHSPCs significantly improves their engraftment in NOD/SCID mice (Brenner et al., 2004; Kahn et al., 2004) and corrected the immediate loss of human HSPCs derived from pluripotent stem cells in NSG mice (Reid et al., 2018). Recently, a knock-in of human *CXCR4* into the mouse gene locus showed competent mouse CXCL12 to human CXCR4 signaling across species (Costa et al., 2018).

Transforming growth factor beta1 (TGFB1), a pleiotropic cytokine that is produced in a latent complexed form by HSCs and progenitor cells (HSPCs), hematopoietic and several different non-hematopoietic niche cells (Table 1), indirectly regulates the responsiveness of mouse HSPCs to mSCF through the decrease of mKIT expression (Dubois et al., 1994). Surgical denervation of *Gfap-GFP^+^* non-myelinating Schwann cells results in loss of HSCs (Yamazaki et al., 2011). These Schwann cells support HSC maintenance most probably through conversion of latent to bioactive mTGFB1 via cell-to-cell contact *in situ* (Yamazaki et al., 2011). Since different concentrations of the three isoforms mTGFB1, −2 and −3 differentially affect differentiation and self-renewal (Langer et al., 2004), it is thought that mTGFB is important in the fine-tuning of HSC cell division (Sitnicka et al., 1996b). Murine and human TGFB1 and its receptor TGFBR1 show a high protein identity between mouse and human (89.7 and 95.7%, Table 1) suggesting possible interspecies cross-reactivity. Indeed, murine TGFB1 and −3 were shown to stimulate hematopoietic specification of differentiating human embryonic stem cells (Ledran et al., 2008). Consistently, in a xenotransplant model treatment of recipient mice with anti-human TGFB1 antibody GC1008 lead to higher engraftment of the human donor cells (Zhang et al., 2016) confirming murine TGFB1 to play a role in huHSPC maintenance.

Another pathway crucial for HSC maintenance and differentiation is the JAGGED – NOTCH interaction. The receptor NOTCH1 is expressed by mouse and human HSPCs (Varnum-Finney et al., 1998) and the ligand JAG1 is expressed by MSCs (Varnum-Finney et al., 1998), osteoblasts (Calvi et al., 2003), and endothelial cells (Poulos et al., 2013; Table 1). In the integrated analysis of single cell RNAseq data (Dolgalev and Tikhonova, 2021), *Jag1* is mostly expressed in AdipoCAR cells, *Jag2* in AECs, *Notch1* in ECs, *Notch2* to -*4* mostly in mesenchymal cells, whereas *Notch3* has the highest expression in AdipoCAR cells. The precise role of NOTCH for murine and human HSC function is still controversially discussed (Lampreia et al., 2017). Among other possibilities it was suggested that NOTCH signaling is exclusively important for lymphoid differentiation (Radtke et al., 1999; Wilson et al., 2001). Consistent with this idea, *Notch1* is found expressed to low levels by purified mouse HSCs (Table 1), whereas committed progenitors, especially common lymphoid progenitors (CLP), express high levels of *Notch1*^2^. NOTCH1 and JAG1 share 90.2 and 96.6% protein sequence identity between mice and humans (Table 1) and NOTCH signaling is highly conserved across species (Artavanis-Tsakonas, 1999). Thus, the JAG1 - NOTCH1 signaling axis presents a promising candidate to mediate huHSPC – mouse niche interactions. HuHSCs treated with hJAG1 show a moderately enhanced engraftment after transplantation into NOD/SCID mice (Karanu et al., 2000). Consistently, huHSCs that bind a JAG1-FLAG protein show higher engraftment levels compared to huHSCs that don’t bind JAG1-FLAG protein (Guezguez et al., 2013). Inhibition of NOTCH signaling in xeno-HSC-engrafted mice led to a reduction of overall chimerism and huHSC frequency (Guezguez et al., 2013). This finding suggests a role for mNOTCH in maintaining huHSCs in the endosteal niche in a xenotransplantation setting. To improve NOTCH signaling in humanized mice, human JAG1 was expressed by osteoblasts in NOG mice [hJ1-NOG (Negishi et al., 2014)]. An increased frequency of huHSCs was found in hJAG1-transgenic recipient mice, suggesting that the maintenance of phenotypic huHSCs is improved through the NOTCH1-hJAG1 signaling axis.

Thrombopoietin (THPO) is a cytokine mainly involved in the expansion and differentiation of megakaryocytes (Kaushansky et al., 1994). Beyond that, THPO was one of the first cytokines identified to support HSPC proliferation *in vitro* (Ku et al., 1996; Sitnicka et al., 1996a) and is still an important component of the cytokine mix used to expand functional HSCs in culture (Wilkinson et al., 2019). Constitutive deletion of *Thpo* or its receptor myeloproliferative leukemia protein (*Mpl*) results in reduced numbers of HSPCs in mice (Qian et al., 2007). Murine THPO maintains adult HSCs in quiescence (Qian et al., 2007; Yoshihara et al., 2007). Initially, it was thought that mTHPO is produced by osteoblasts (Yoshihara et al., 2007), however, recent analysis suggests that mTHPO rather is provided by parenchymal liver cells (*Alb-cre^+^* hepatocytes) but not by hematopoietic cells, osteoblasts, or *Lepr*^+^ MSCs in mice (Decker et al., 2018). THPO and MPL show considerable low protein identity between mouse and human (71.6 and 80.6%, respectively, Table 1), which is why they may act cross-reactive between species only when used at supraphysiological doses *in vitro* (Rongvaux et al., 2011). Consistently, endogenous mTHPO provides only insufficient support for the engraftment of donor human HSCs, and the injection of hTHPO improves the engraftment of huHSCs (Verstegen et al., 2003). Further, the knock-in of human *THPO* into BRg mice (BRg-*TPO*^*h/h*^) induces increased huHSC engraftment on the expense of the maintenance of endogenous muHSCs and megakaryopoiesis (Rongvaux et al., 2011), confirming suboptimal interspecies cross-reactivity.

TEK (TIE2) is a receptor tyrosine kinase mainly expressed by endothelial cells (Sato et al., 1993) and HSCs (Iwama et al., 1993). Constitutive depletion of mTEK results in embryonic lethality with lack of HSPCs and decreased vascular networks, displaying the importance of mTEK-mediated signals during development (Takakura et al., 1998). TEK is specifically expressed by quiescent HSCs (Arai et al., 2004) that contain the majority of the repopulation potential compared to TEK-negative HSCs of murine (Ito et al., 2016) or human (Yuasa et al., 2002) origin. Angiopoietin-1 (ANG1) and ANG2 bind to TEK and treatment of mouse HSCs with ANG1 improves their engraftment, whereas ANG2, in contrast, antagonizes the beneficial effect of ANG1 (Gomei et al., 2010). ANG1 is moderately expressed by murine osteoblasts (Arai et al., 2004) and to higher levels by *Lepr*^+^ MSCs (Zhou et al., 2015; Table 1), but also by HSCs and megakaryocytes (Zhou et al., 2015). TEK^+^ HSCs are found in close proximity to ANG1^+^ osteoblasts (Arai et al., 2004). Protein identity between mouse and human TEK and ANG1 is 92.7 and 97.4% (Table 1), respectively, suggesting a molecular communication between murine osteoblasts and huHSC via this axis. However, conditional depletion of mANG1 from MSCs or osteoblasts has no effect on HSC numbers and function (Zhou et al., 2015). Furthermore, HSC-, megakaryocyte-specific, or even constitutive deletion of *Ang1* has also no effect on HSC biology, suggesting functional compensation through alternative ligands (Zhou et al., 2015). Taken together, although the direct functional importance for TEK on murine and human HSCs remains to be shown, TEK expression corresponds with improved human and mouse HSC repopulation activity independent of ANG1 mediated communication *in vivo*.

The phosphorylated glycoprotein Osteopontin (OPN or secreted phosphoprotein 1, SPP1), is expressed by MSCs, osteoblasts, and endothelial cells (Table 1) with the highest expression by AdipoCAR cells (Dolgalev and Tikhonova, 2021). After cleavage by thrombin (Grassinger et al., 2009) it binds to a variety of matrix receptors, including CD44 and very late antigen-4 (VLA4, integrin a4b1, both highly expressed on HSCs, Table 1). VLA4 on HSPCs further binds vascular cell adhesion molecule 1 (VCAM1) that is highly expressed by stromal reticular cells and endothelial cells lining bone marrow sinusoids (Jacobsen et al., 1996; Table 1) and integrin engagement mediates intimate cell-to-cell contact required for the support or HSPC function (Jung et al., 2005). Consistently, the conditional deletion of *Itga4* from adult HSPCs (Scott et al., 2003) or *Vcam1* from endothelial cells (Koni et al., 2001) mobilizes HSPCs to the blood and impairs homing to the bone marrow niche, critically delaying short-term engraftment after transplantation (Koni et al., 2001; Scott et al., 2003). Constitutive deletion of *Opn* in mice results in elevated HSC numbers and better engraftment of mOPN-deficient donor BM cells after transplantation (Stier et al., 2005). Consistently, the transfer of wild type cells into an mOPN-deficient microenvironment impairs donor HSC engraftment (Nilsson et al., 2005), which may be based on the efficient chemoattractant function of mOPN (Grassinger et al., 2009). However, OPN (63.3%) and its receptors (CD44 67.2%, VLA4 85%) show moderate protein identity between mouse and human (Table 1), suggesting a minor contribution to the maintenance of human HSPCs in the murine niche.

Fibroblast growth factors (FGFs) make up an extensive family of 23 growth factors that regulate a wide range of biological processes during development and in the adult organism (Turner and Grose, 2010). Two members, FGF1 and FGF2, are suggested to play a role in hematopoiesis. *In vitro*, FGF1 supports expansion of functional murine HSPCs (de Haan et al., 2003; Yeoh et al., 2006). Further, the corresponding receptor, FGFR1, is a marker enriching long-term repopulating activity in mice (de Haan et al., 2003). Deletion of *Fgfr1* on HSPCs renders HSCs intact in the steady state but impairs the recovery of HSPCs after cytotoxic insult and delays repopulation of irradiated recipient mice (Zhao et al., 2012). Megakaryocytes are producers of FGF1 during post-injury regeneration and, consistently, conditional deletion of *Fgf1* from megakaryocytes impairs HSC regeneration after 5FU treatment (Zhao et al., 2014). There is high protein identity between human and mouse FGF1 and FGFR1, 95.5 and 98.4%, respectively, indicating possible cross-species reactivity (Table 1). Together with transcript detection in endothelial cells and MSCs (Table 1), the interaction of mFGF1 and hFGFR1 might be of importance during engraftment of human HSPCs in the murine bone marrow. FGF2 exerts indirect effects on HSPCs by acting on other niche cell populations. FGF2 expands HSPCs within bone marrow cultures but fails to do so when sorted HSPCs are cultured “alone” (Zhao et al., 2012). The anabolic effects of PTH (Calvi et al., 2003), including expansion of osteoblasts and MSCs are highly dependent on FGF2 (Sabbieti et al., 2009). Treatment of mice with FGF2 leads to an expansion of *Nestin*^+^ and other stromal cells, which in turn produce increased levels of SCF mediating an expansion of HSPCs (Itkin et al., 2012). FGF2 levels are also increased after challenges including chemotherapy or irradiation, indicating a role in emergency hematopoiesis. These data indicate that FGF2 affects HSPCs by remodeling the HSC niche, however, *Fgf2* is not expressed by non-hematopoietic niche cells analyzed in this review.

Consistent with its pleiotrophic functions, the small cytokine pleiotrophin (PTN, also known as osteoblast-specific factor-1, OSF-1, or heparin-binding growth-associated molecule, HB-GAM) plays important roles in the proliferation and differentiation in various cell types. While its absence does not affect HSCs in steady-state hematopoiesis, PTN was shown to improve hematopoietic regeneration after sublethal irradiation (Himburg et al., 2010; Istvanffy et al., 2011). As discussed before for SCF, the specific niche cell type producing PTN is of great importance for its functional effects (Himburg et al., 2018). *Lepr*^+^ MSCs are the key source of mPTN during steady-state, express it constitutively and *Ptn* deletion from MSCs reduces HSC numbers. mPTN is also expressed by ECs but endothelial cell-specific depletion has no effect on the HSC pool. However, PTN production by endothelial cells is massively increased after myeloablation and its deletion impairs hematopoietic regeneration that is unaffected by MSC-specific *Ptn* deletion (Himburg et al., 2018). Further, overexpression of hPTN in mice increases bone formation, fracture healing, and bone repair (Li et al., 2005). hPTN produced by human tonsil-derived MSCs as well as recombinant hPTN was shown to enhance engraftment of mouse HSCs after prior cultivation (Kim et al., 2020). Engraftment capacity of *ex vivo* hPTN-treated huHSPCs in NSG mice was also greatly increased compared to cultivation with SCF, FLT3L, and TPO alone (Himburg et al., 2010). These findings, together with the very high protein identity between mouse and human PTN (97.5%, Table 1) point toward possible cross-species support of huHSCs by murine niche PTN.

The interaction between Granulocyte-Colony Stimulating Factor (GCSF, CSF) and CSF3 receptor (CSF3R, GCSFR) is important for granulocyte differentiation, and the constitutive deletion of either the ligand [(Lieschke et al., 1994) mouse; (Dong et al., 1994) human] or its receptor (Liu et al., 1996; Mitsui et al., 2003) shows the crucial role in neutrophil production. In mice, CSF3 secreted by endothelial cells (Boettcher et al., 2014; Table 1) is important for emergency granulopoiesis in response to bacterial challenges through the increase of CSF3-responsive progenitors (Boettcher et al., 2014). In humans, the proliferative response of CD34^+^ HSPCs to osteoblast-derived CSF3 also suggests an expansion on the level of stem or progenitor cells (Taichman and Emerson, 1994). Exogenous CSF3 administration mobilizes murine (Molineux et al., 1990) and human (Uyl-de Groot et al., 1994; Lévesque et al., 2003) HSCs to the periphery through the remodeling of the stem cell niche reducing the number of osteoblasts and macrophages (Winkler et al., 2012). CSF3 also regulates HSC activity through the activation of toll-like receptor signaling (Schuettpelz et al., 2014). While human recombinant CSF3 efficiently activates huHSCs in NOD/SCID mice (Broxmeyer et al., 2005), the precise role of endothelial cell-derived murine CSF3 in huHSC engraftment is unknown. Based on the low protein identity between human and mouse CSF3 and CSF3R (Table 1) we hypothesize that this interaction is of minor importance for the maintenance of huHSCs in mice.

Duffy antigen receptor for chemokines (DARC or CD234, encoded by atypical chemokine receptor 1, *Ackr1*) is a transmembrane protein produced by sinusoidal ECs (Bandyopadhyay et al., 2006; Table 1), macrophages (Hur et al., 2016) and (nucleated) erythroid precursors (Duchene et al., 2017). DARC directly interacts with CD82 that is predominantly expressed by LT-HSCs (Hur et al., 2016). DARC-deficiency in erythroid precursors decreases the myeloid output of murine HSPCs and leads to phenotypically distinct neutrophils that leave the circulation and cause neutropenia in mice and human (Duchene et al., 2017). Further, macrophage-derived mDARC promotes HSC quiescence via TGFB1 signaling (Hur et al., 2016). This mechanism also seems to support huHSC maintenance *in vitro* (Hur et al., 2016). Given that RBC but not leukocyte transfusions prevent bleeding-dependent HSC expansion in mice (Cheshier et al., 2007) it is tempting to speculate that DARC on RBC precursors may also induce quiescence in mHSCs. However, given the low protein sequence identity between mouse and human DARC it remains to be shown whether mDARC produced by murine macrophages or erythroid cells can support huCD82-positive huHSCs in humanized mice.

## Concluding Remarks

It is an exciting time for research about the interrelationship of HSCs and their BM niche. We have here attempted to review decades of studies describing how changes in BM niche composition (ecology) and structure (remodeling) modulate hematopoiesis. With the technical advancements of the last decade in RNAseq, genomic targeting, and imaging techniques and how these types of data are analyzed it is slowly becoming apparent which populations in the BM are important for early and late hematopoiesis and which factors they produce for their hematopoiesis-promoting activity. It would be too simple to attribute support of *in vivo* hematopoiesis solely to AdipoCAR (MSPC-Adipo, *Lepr*^+^*Adipoq*^+^*Cxcl12*^+^ cells) and ECs (either sinusoidal, arterial, or arteriolar). Indeed, whereas these two cell types produce the bulk of the known hematopoiesis promoting factors (Table 1), other factors known to be required for HSC maintenance in steady-state, aging, or transplantation settings are clearly expressed by other cell types, such as OsteoCAR cells and other osteoblasts, chondrocytes and fibroblasts. Interestingly, the above reviewed studies also reveal the cross-species reactive factors which enable engraftment and maintenance of human HSPCs in mice, thus giving unique insights in the requirements for species-specific and -cross-reactive activities. The most important development surrounding BM niche composition and remodeling is that the in-depth analyses start to reveal the secrets of the BM niche enabling the identification of possible targets to maintain or restore the microenvironment required for long-term maintenance of healthy HSC, as well as ways to interrupt and perhaps even eradicate their malignant counterparts.

## Author Contributions

JF, CS, ME, CW, and RO edited the manuscript. JF, CW, and RO prepared the figures and table. JF, TL, GP, CS, SR, AO, DN, ME, CW, and RO wrote parts of the manuscript. All authors contributed to the article and approved the submitted version.

## Conflict of Interest

The authors declare that the research was conducted in the absence of any commercial or financial relationships that could be construed as a potential conflict of interest.

## Footnotes

1 https://singlecell.broadinstitute.org/single_cell/study/SCP1248

2 www.immGen.org

## Abbreviations

5FU: 5-fluorouracil
AEC: arterial endothelial cell
AML: acute myeloid leukemia
BM: bone marrow
CAR: CXCL12-abundant reticular (cell)
DTR: diphtheria toxin receptor
EC: endothelial cell
ECM: extracellular matrix
EMCN: endomucin
ER: endoplasmic reticulum
GvHD: graft-versus-host disease
HPC: hematopoietic progenitor cell
HPSC: hematopoietic stem and progenitor cell
HSC: hematopoietic stem cell
huHSC: human HSC
LPS: lipopolysaccharide
MDS: myelodysplastic syndrome
MPN: myeloproliferative neoplasm
MSC: mesenchymal stromal cell
MSPC: mesenchymal stem and progenitor cell
P4H: prolyl-4-hydroxylase
PaS: PDGFRa^+^ Sca-1^+^ (cell)
pIpC: polyInosine-polyCytosine
ROS: reactive oxygen species
SEC: sinusoidal endothelial cell.
